# A new measure for the revised reinforcement sensitivity theory: psychometric criteria and genetic validation

**DOI:** 10.3389/fnsys.2015.00038

**Published:** 2015-03-16

**Authors:** Martin Reuter, Andrew J. Cooper, Luke D. Smillie, Sebastian Markett, Christian Montag

**Affiliations:** ^1^Department of Psychology, University of BonnBonn, Germany; ^2^Laboratory of Neurogenetics, University of BonnBonn, Germany; ^3^Center of Economics and Neuroscience, University of BonnBonn, Germany; ^4^Department of Psychology, Goldsmiths, University of LondonLondon, UK; ^5^Melbounre School of Psychological Sciences, The University of MelbourneMelbourne, VIC, Australia; ^6^Department of Psychology, University of UlmUlm, Germany

**Keywords:** reinforcement-sensitivity-theory, anxiety, fear, revised RST questionnaire, AVPR1a, rs11174811

## Abstract

Jeffrey Gray's Reinforcement Sensitivity Theory (RST) represents one of the most influential biologically-based personality theories describing individual differences in approach and avoidance tendencies. The most prominent self-report inventory to measure individual differences in approach and avoidance behavior to date is the BIS/BAS scale by Carver and White (1994). As Gray and McNaughton (2000) revised the RST after its initial formulation in the 1970/80s, and given the Carver and White measure is based on the initial conceptualization of RST, there is a growing need for self-report inventories measuring individual differences in the revised behavioral inhibition system (BIS), behavioral activation system (BAS) and the fight, flight, freezing system (FFFS). Therefore, in this paper we present a new questionnaire measuring individual differences in the revised constructs of the BIS, BAS and FFFS in *N* = 1814 participants (German sample). An English translated version of the new measure is also presented and tested in *N* = 299 English language participants. A large number of German participants (*N* = 1090) also filled in the BIS/BAS scales by Carver and White (1994) and the correlations between these measures are presented. Finally, this same subgroup of participants provided buccal swaps for the investigation of the arginine vasopressin receptor 1a (AVPR1a) gene. Here, a functional genetic polymorphism (rs11174811) on the AVPR1a gene was shown to be associated with individual differences in both the revised BIS and classic BIS dimensions.

## Introduction

The Reinforcement Sensitivity Theory (RST) of personality has in recent years become one of the most prominent biologically oriented theories in personality psychology (Corr, 2008; Smillie et al., 2011). At the core of the classic form of this theory are the behavioral activation and behavioral inhibition systems (BAS and BIS, respectively). These systems regulate approach toward appetitive stimuli and avoidance/withdrawal of aversive stimuli. Individual differences in the functioning of the BIS and BAS are thought to provide the biological foundation for complex personality traits (see also Montag et al., 2013). Gray proposed that the BAS is anchored in mesolimbic dopaminergic pathways (e.g., Pickering and Gray, 2001), thereby sharing similar ideas with Panksepp's SEEKing system (Panksepp and Moskal, 2008) and Depue's Behavioral Faciliation System (e.g., Depue and Collins, 1999). Here, mesolimbic dopamine function is thought to underpin energized approach behavior toward appetitive stimuli (Schultz, 2007). Individuals with stronger dopaminergic firing in these brain regions might be characterized as full of energy, having a tendency toward outgoing explorative behavior, and being more motivated to pursue rewards (e.g., Leyton et al., 2002). In contrast, individuals with a more reactive BIS might be characterized as more anxious, avoidant, and more motivated to avoid threat or punishment^1^. According to the original conceptualization of RST, the BIS is hypothesized to be anchored around a core network comprising the septo-hippocampal-system (e.g., Gray, 1982).

A major revision to RST has resulted in a somewhat updated understanding of the systems described above (Gray and McNaughton, 2000; McNaughton and Corr, 2004), with particularly notable implications for the role of the BIS and what has now been termed the Fight Flight Freezing system (FFFS, reflecting Fear^2^). The first major change from the “classic” to the “revised” RST is the removal of the distinction between conditioned and unconditioned stimuli (see McNaughton and Corr, 2004). In the classic version of the RST, the BAS and BIS were thought to be activated only by conditioned rewarding and punishing stimuli, respectively. In the revised RST, the BAS is proposed to be responsive to *all* rewarding and appetitive stimuli, while the FFFS is proposed to be responsive to *all* punishing and threatening stimuli. Conversely, the BIS is now thought to be activated by instances of goal conflict, such as when a threatening stimulus must be approached, or when mixed signals of reward and punishment are present. In rodent models, such conflict is well represented by a rodent being placed in an experimental setting prepared with cat odor. While the visual information clearly indicates that no cat is in close proximity, the olfactory senses of the rodent suggest otherwise. Such experiments have been conducted in a setting called the visible burrow system by Blanchard and Blanchard (1989) and Blanchard et al. (1993, 2001), among others. Activation of the BIS is still equated with the experience of anxiety, although this is now attributed to goal conflict, and is grounded in the “concurrent activity in the amygdala and septo-hippocampal system” (Gray and McNaughton, 2000, pp. 122–123). Observable behavior accompanying BIS activation is thought to include careful and slow approach behavior toward the potentially dangerous stimuli, and risk assessment behaviors (e.g., visual scanning of the environment). This careful approach behavior in a potentially dangerous situation is important, because it can generate new information to help solve the conflict (is a cat near, or not?) resulting either in activation of the BAS (hence, exploration behavior e.g., to seek for food), or activation of the FFFS, triggering withdrawal behavior such as fight, flight or freezing.

As outlined above, both the hippocampus and the amygdala have been outlined as playing an important role for the BIS (Gray and McNaughton, 2000; pp. 122–123). This idea has already received support from studies in the human neuroscience literature. For example, a study by Barrós-Loscertales et al. (2006) observed a positive correlation between gray matter volumes of the hippocampus and amygdala, and scores on a questionnaire measuring BIS reactivity. Supporting these findings, Cherbuin et al. (2008) were also able to observe a positive correlation between BIS scores and hippocampus volume. Of note, these studies administered self-report inventories which were originally developed to measure the classic BIS and BAS dimensions. Moreover, a review by our own group showed that similar personality constructs, such as Neuroticism or Harm Avoidance^3^, have also been linked to the hippocampus, but in the opposite direction—that is, a negative association between negative emotionality and gray matter volume in structures of the temporal lobe is also plausible (Montag et al., 2013).

One of the most pressing issues in personality psychology when dealing with the revised RST is the measurement of individual differences in the BIS (reflecting anxiety) and the FFFS (reflecting fear) in terms of the changes made to the theory. There already exists a first questionnaire, the *Reinforcement Sensitivity Questionnaire (RSQ)*, measuring the revised RST, but the items were only published in a Serbian book chapter and not in English language. However, the authors have published an article on validation data of their RSQ where the principles of the questionnaire construction are described (Smederevac et al., 2014). In the RSQ the BAS is conceptualized with a focus on behaviors indicating sensitivity to signals of reward rather than on those indicating sensitivity to reward. The BIS is defined as conflict between worries (arising from the scanning of internal resources) and the outcome/feedback of real situations. The FFFS is represented by three distinct scales: Fight items include aggressive reactions to the emotion of fear caused by present threats, Freeze items express the inability to articulate necessary verbal responses to threat and Flight is defined as reaction to real danger which can be avoided. It is important to mention that the questionnaire construction and data collection of the RSQ and the *Reuter and Montag rRST-Q* happened parallel in time so that we had no chance to profit from the ideas and results of Smederevac et al. Theoretical overlap and difference between the RSQ and our rRST-Q are described in the Discussion section.

On this basis, the first aim of the present study is to provide a new measurement tool for the revised RST, with a particular focus on disentangling measurement of the BIS and FFFS constructs. In this initial investigation, we employ molecular genetic methods to investigate the validity of our new measure.

Of particular importance to the present study is that the emotion of anxiety is influenced by a large number of neurotransmitters, including gamma amino butyric acid (GABA; e.g., Nemeroff, 2002), and classic monoamines such as dopamine (e.g., Montag et al., 2012) and serotonin (Lesch et al., 1996). In addition to these classic “anxiety molecules,” recent years have seen a rise in studies investigating neuropeptides such as oxytocin and arginine vasopressin (AVP) to help better understand negative emotionality and personality (for an overview see Montag and Reuter, 2014). The nonapeptide oxytocin has become a major research focus as it seems to play an important role in social cognition and has been associated with trust behavior (Kosfeld et al., 2005). Kirsch et al. (2005) demonstrated that the nasal administration of oxytocin reduces amygdala activity while processing pictures depicting unpleasant content. Similar effects have been observed by Domes et al. (2007), who reported a down-regulation of the amygdala while processing emotional faces after administration of oxytocin. As noted above, Kosfeld et al. (2005) found that meeting a trusted person could trigger oxytocin secretion, which results in the dampening of alarm signals usually elicited by the amygdala when encountering a stranger. Therefore, oxytocin could be of particular relevance for understanding the emotion of (social) anxiety, elicited by uncertainty when meeting and engaging with strangers.

There has been even less social neuroscience research on the role of the nonapeptide vasopressin in the context of social anxiety. The initial studies in this area with humans point toward an equally important role for vasopressin in social cognition (Zink and Meyer-Lindenberg, 2012). Of particular importance is the study by Zink et al. (2010), reporting that the subgenual anterior cingulate cortex was more strongly activated under the influence of vasopressin, compared to placebo, when humans participated in a classic face matching paradigm using fearful and angry faces. During fear processing strong subcortical signals can be observed, putatively due to a lack of inhibition by the prefrontal cortex (Mobbs et al., 2007). Arginine-Vasopressin (AVP) could counteract these fear (or anxiety) effects by strengthening the PFC activity as a top-down fear/anxiety regulator. Further evidence for a role of AVP in anxiety/fear comes from animal research. Among others, Appenrodt et al. (1998) observed that the administration of AVP in septal regions attenuates anxiety-related behavior in the form of longer time spent in the open arms of the elevated plus maze test in rats. The major target of AVP for cell signaling is the arginine vasopressin receptor 1 a (AVPR1a). Consequently, knocking out the gene coding for AVPR1a could be associated with a reduction in anxiety, as measured by the elevated plus maze test mentioned above (Egashira et al., 2007). In order to translate these interesting findings to humans, molecular genetic association studies have already investigated genetic variants on the AVPR1A and AVPR1B^4^ genes in relation to individual differences in a vast range of human behaviors, including anxiety related personality traits (Meyer-Lindenberg et al., 2009; Kazantseva et al., 2014), altruistic behavior (Avinun et al., 2011), musical aptitude (Ukkola et al., 2009), pair bonding (Walum et al., 2008), and autism (Yirmiya et al., 2006; Yamasue, 2013).

In the present study, we hypothesized that genetic variation on the AVPR1a gene would be related to individual differences in measures of BIS (both the classic and revised form), but should not be associated with our measure of FFFS. We focused on the single nucleotide polymorphism (SNP) rs11174811 on the AVPR1a gene (located on chromosome 12q), because not only has it been associated with phenotypes related to anxiety/negative emotionality [e.g., stress reactivity, drug addiction, blood pressure, partnership satisfaction, and aggressive behavior (Maher et al., 2011; Nossent et al., 2011; Levran et al., 2014; Malik et al., 2014)], but also the functionality of this gene variant has been demonstrated by means of mRNA expression in postmortem brain tissue. Expression levels in samples homozygous for the major C-allele (genotype CC) were significantly lower than in samples with at least one minor A-allele (genotypes AA or CA; Maher et al., 2011).

In sum, the main aims of the present research are as follows: First, we report on the development of a new questionnaire measuring individual differences in the revised constructs of Gray and McNaughton's BAS, BIS and FFFS dimensions. Second, this new questionnaire, called *Reuter and Montag's rRST-Q*, is cross-validated against the widely used *Carver and White BIS/BAS* scales, in order to examine the convergent and divergent validity of the new questionnaire. We would expect only moderate correlations between the same constructs measured across the two self-report-inventories given the differences between the classic and revised models of RST, as outlined above. We would also expect only low to moderate correlations between the revised FFFS measure in the rRST-Q and the BIS scale from the Carver and White scale, on the same basis. The third aim of the study was to examine whether a genetic variant is associated with individual differences in the BIS. As AVP has been understudied in the context of anxiety (although the first evidence points toward such an association, as outlined above) so far, we tested for a link between rs11174811 and the BIS, as measured by both the Reuter and Montag and the Carver and White scale. Given the small number of studies dealing with the functional polymorphism on the AVPR1a gene in the context of negative emotionality, we have not provided a directional hypothesis for this potential effect.

## Methods

### Participants

The results of this study will be presented across three sections. The German version of the *rRST-Q* reported on in the first section of the results was completed by *N* = 1814 participants (*n* = 704 males and *n* = 1110 females, mean-age: 24.86, *SD* = 7.28). The English translated version of the *rRST-Q*, also reported on in this first section, was filled in by *N* = 299 participants (*n* = 79 males and *n* = 220 females, mean-age: 24.12, *SD* = 8.49). The participants were predominantly university students in both the German and English samples. In the last two sections of the results, *N* = 1090 participants (*n* = 325 males and *n* = 765 females; mean-age: 25.27, *SD* = 8.09), from the German sample described above, filled in *Reuter and Montag rRST-Q* as well as *Carver and White's (1994) BIS/BAS* scales, and also provided buccal swaps for genotyping a genetic variation of the AVPR1a gene. The study was approved by the psychology ethics committee of the University of Bonn, Germany.

### Measures

Two questionnaires were administered to measure individual differences in RST-relevant personality constructs. We administered a new questionnaire called *Reuter and Montag's rRST-Q* to measure individual differences in the revised BAS, BIS, and FFFS constructs. Additionally, most of the German participants also completed the most widely used RST self-report measure, the *Carver and White BIS/BAS* scale, developed using the original RST model.

### Reuter and Montag's rRST-Q

*Reuter and Montag's rRST-Q* consists of 31 items, with a four point Likert scale ranging from “strongly disagree” to “strongly agree.” The BAS is measured by eight items, the BIS by 11 items and the FFFS by 12 items. The original item pool for the rRST-Q consisted of 34 items; three items were excluded during the development process to improve both the factor structure and the internal consistencies of the scale. The German version of the scale was translated into English by a bilingual German-English speaker; the translated items were then checked by a native English speaker and some minor modifications were made to several of the items. This version was then back-translated to German by a different bilingual German-English speaker, and the resultant back-translated German items were checked against the original German items for consistency. Tables 1, 2 present all items from *Reuter and Montag's rRST-Q* in German and English.

**Table 1:** **German version of *Reuter and Montag's rRST-Q*; Likert scaling: ① trifft für mich gar nicht zu, ② trifft für mich eher nicht zu, ③ trifft für mich eher zu, ④ trifft für mich genau zu**.

1. Ich bin ein spontaner Mensch. (rBAS)	①	②	③	④
2. Oft bin ich froh, wenn mir eine Entscheidung abgenommen wird. (rBIS)	①	②	③	④
3. In bedrohlichen Situationen bin ich oftmals wie gelähmt. (FFFS—Freezing)	①	②	③	④
4. Oftmals zweifele ich, ob sich der Einsatz für eine Sache lohnt. (rBIS)	①	②	③	④
5. Ich bin meist voller Tatendrang. (rBAS)	①	②	③	④
6. Bei Gefahr tendiere ich dazu, die Flucht zu ergreifen. (FFFS—Flight)	①	②	③	④
7. Wenn ich die Wahl zwischen zwei attraktiven Möglichkeiten habe, tue ich mich mit meiner Entscheidung schwer. (rBIS)	①	②	③	④
8. Meine Freunde würden mich eher für einen unentschlossenen Menschen halten. (rBIS)	①	②	③	④
9. Auch eher unangenehme Aufgaben gehe ich meist ohne zu zögern an. (FFFS—Freezing) **R**	①	②	③	④
10. Ich lasse unangenehme Termine gerne verstreichen. (FFFS—Freezing)	①	②	③	④
11. Unsicherheit kann ich nur schwer ertragen. (rBIS)	①	②	③	④
12. Ich gehe öfters ein Risiko ein. (rBAS)	①	②	③	④
13. Ich bin für neue Dinge leicht zu begeistern. (rBAS)	①	②	③	④
14. Unangenehme Dinge sitze ich gerne aus. (FFFS—Freezing)	①	②	③	④
15. Wenn ich kritisiert werde, bin ich meist unfähig, mich zu verteidigen. (FFFS—Fight) **R**	①	②	③	④
16. Um Schlimmeres zu vermeiden, gebe ich lieber klein bei. (FFFS—Fight) **R**	①	②	③	④
17. Angriff ist die beste Verteidigung. (FFFS—Fight)	①	②	③	④
18. Nur wer wagt, gewinnt. (rBAS)	①	②	③	④
19. Konfrontationen gehe ich für gewöhnlich aus dem Weg. (FFFS—Flight)	①	②	③	④
20. Erkenne ich, dass ein negatives Ereignis unvermeidbar ist, versetzt mich dies in Panik. (FFFS—Flight)	①	②	③	④
21. Im Restaurant habe ich keine Probleme, mich für ein Gericht zu entscheiden. (rBIS) **R**	①	②	③	④
22. Ich bin ein eher schlagfertiger Mensch. (FFFS—Fight)	①	②	③	④
23. Oft weiß ich nicht, was ich will. (BIS)	①	②	③	④
24. Wenn ich die Chance sehe, etwas zu erreichen, bin ich sofort Feuer und Flamme. (rBAS)	①	②	③	④
25. Ich bin ein kontaktfreudiger Mensch. (rBAS)	①	②	③	④
26. Muss ich mich zwischen zwei unangenehmen Alternativen entscheiden, fällt mir die Wahl des “kleineren” Übels eher schwer. (rBIS)	①	②	③	④
27. Ich beharre im Allgemeinen auf meinen Rechten. (FFFS—Fight)	①	②	③	④
28. Oft fühle ich mich hin und her gerissen. (rBIS)	①	②	③	④
29. Eine schwere und wichtige Prüfung bereitet mir im Voraus große Sorgen. (rBIS)	①	②	③	④
30. Wichtige Entscheidungen schiebe ich oftmals vor mir her. (rBIS)	①	②	③	④
31. Bietet sich mir eine gute Gelegenheit, ergreife ich diese, ohne zu zögern. (rBAS)	①	②	③	④

R, reversed item.

Note: The total FFFS scale score is comprised of high Flight/Freezing and low Fight scores. Therefore, high scores on Fight reflect low fear. So when calculating a total for the FFFS scale, the items for Fight 15**R**,16**R**, 17, 22 need to be reversed, resulting in 15, 16, 17**R**, and 22**R**.

**Table 2:** **English version of *Reuter and Montag's rRST-Q;* Likert scaling: ① strongly disagree, ② disagree, ③ agree, ④ strongly agree**.

1. I'm a spontaneous person. (rBAS)	①	②	③	④
2. I'm often glad if someone makes decisions for me. (rBIS)	①	②	③	④
3. I often feel paralyzed when in a dangerous situation. (FFFS—Freezing)	①	②	③	④
4. I often doubt if my efforts will pay off. (rBIS)	①	②	③	④
5. Most of the time I have a thirst for action. (rBAS)	①	②	③	④
6. When faced with danger, I tend to flee. (FFFS—Flight)	①	②	③	④
7. If I have the choice between two appealing options, I have difficulty deciding on one. (rBIS)	①	②	③	④
8. My friends think of me as an indecisive person. (rBIS)	①	②	③	④
9. I usually approach unpleasant tasks without hesitation. (FFFS—Freezing) **R**	①	②	③	④
10. I will gladly let unpleasant tasks slip by. (FFFS—Freezing)	①	②	③	④
11. I find it hard to bear uncertainty. (rBIS)	①	②	③	④
12. I often take risks. (rBAS)	①	②	③	④
13. I'm easily inspired by new things. (rBAS)	①	②	③	④
14. I like sitting unpleasant things out. (FFFS—Freezing)	①	②	③	④
15. Most of the time, I cannot defend myself if I am criticized. (FFFS—Fight) **R**	①	②	③	④
16. To avoid worse things happening, I would rather give in. (FFFS—Fight) **R**	①	②	③	④
17. Attack is the best form of defense. (FFFS—Fight)	①	②	③	④
18. Whoever dares wins. (rBAS)	①	②	③	④
19. I usually avoid confrontations. (FFFS—Flight)	①	②	③	④
20. When an unpleasant event is inevitable, I'm thrown into a state of panic. (FFFS—Flight)	①	②	③	④
21. I don't have problems deciding on a dish in a restaurant. (BIS) **R**	①	②	③	④
22. I am a rather quick-witted person. (FFFS—Fight)	①	②	③	④
23. I often don't know what I want. (rBIS)	①	②	③	④
24. I get fired up when I see the chance to achieve something. (rBAS)	①	②	③	④
25. I am an outgoing person. (rBAS)	①	②	③	④
26. When faced with two unpleasant alternatives, it is difficult for me to decide on the lesser of two evils. (rBIS)	①	②	③	④
27. In general, I stand up for myself. (FFFS—Fight)	①	②	③	④
28. I often feel torn between two options. (rBIS)	①	②	③	④
29. I worry greatly before a difficult or important test. (rBIS)	①	②	③	④
30. I usually carefully weigh up the options before making important decisions. (rBIS)	①	②	③	④
31. When offered a good opportunity, I take it without hesitating. (rBAS)	①	③	③	④

*R, reversed item*.

*Note: The total FFFS scale score is comprised of high Flight/Freezing and low Fight scores. Therefore, high scores on Fight reflect low fear. So when calculating a total for the FFFS scale, the items for Fight 15**R**, 16**R**, 17, 22 need to be reversed, resulting in 15, 16, 17**R**, and 22**R***.

The following theoretical considerations form the basis for the construction of *Reuter and Montag's rRST-Q*:

#### Revised BAS

Higher BAS activity should be associated with energetic arousal and approach behavior toward appetitive stimuli, consistent with the BAS and similar systems being described as a “Go get it!” system (Panksepp, 1998). The BAS dimension in this scale has item content measuring approach and goal-directed behavior; those who score high on this BAS scale could be described as bold, adventurous and may show stronger energy and drive when approaching appetitive stimuli.

#### Revised BIS

Higher BIS activity should reflect responses to goal conflict and situations of uncertainty, including hesitation, risk assessment or wary behavior. As proposed by Gray and McNaughton (2000), three kinds of conflict are possible in principle (i.e., approach–approach, approach–avoidance, and avoidance–avoidance). Individuals with a more reactive BIS will tend to have difficulty making decisions when two equally attractive or unattractive options are presented and one option needs to be chosen (e.g., in situations where conflict is apparent).

#### Revised FFFS

In revised RST, the FFFS is associated with three kind of defensive or avoidant responses, namely Fight, Flight, and Freezing. Accordingly, in the *rRST-Q*, high overall trait FFFS is characterized by low fight, high flight and high freezing behavior. This may appear at odds with the notion of defensive attack (e.g., fight behavior) as a classic fear response, as observed in nearly all mammalian organisms. However, in the revised RST fight behavior is only observable if the distance between predator and prey is close to zero, leaving no option for flight or freezing. The probability of such situations occurring for human beings is extremely low (Corr et al., 2013). Furthermore, particularly fearful individuals are perhaps least likely to find themselves in a situation in which there is zero distance between them and a source of threat. As a consequence, and in line with the notion that activity of the FFFS is associated with withdrawal behavior in broad terms, we would characterize a high trait FFFS individual as high in flight and freezing behavior, but a low scorer on fight behavior. A person who is not willing to fight when being attacked might typically withdraw more quickly from unpleasant situations, compared to a person who is more willing to fight when threatened. Clearly when filling in a questionnaire such as this, asking a person to reflect on his or her behavior can only represent an indirect approach to understanding subcortical brain activity in the brain systems of the revised RST. In addition, operationalizing the FFFS as described here putatively leads to positive inter-correlations between all three FFFS subscales.

### *Carver and White BIS/BAS* scale

The *Carver and White BIS/BAS* scale consists of 24 items. The BIS scale consists of seven items and the BAS scales comprise thirteen items. The BAS scale can be split in to three subscales: BAS drive (four items), BAS fun seeking (four items) and BAS reward responsiveness (five items). Four filler items are presented to participants, but not analyzed. The German translation of the *Carver and White BIS/BAS* scale by Strobel et al. (2001) was administered to the German participants in this study. The internal consistencies for the scales derived from the present data set (and contrasted with the data presented by Strobel et al., 2001) are presented in the Results section (see **Table 5**).

### Genetic analyses

DNA was extracted from buccal cells. Automated purification of genomic DNA was conducted by means of the MagNA Pure® LC system using a commercial extraction kit (MagNA Pure LC DNA isolation kit; Roche Diagnostics, Mannheim, Germany). Genotyping of the AVPR1a SNP rs11174811 was performed by means of MALDI-TOF (Matrix-Assisted Laser Desorption/Ionization—Time of Flight) mass spectrometry using The Sequenom MassARRAY® system (Agena Bioscience).

## Results

The Results section of the study is split into three sections. The first section presents descriptive data as well as psychometric data (reliabilities) for both a German and an English version of *Reuter and Montag's rRST-Q*. In addition, confirmatory factor analyses (CFAs) are presented that test the revised RST model (e.g., its factor structure). The second section of the results reports correlations between the German version of the *rRST-Q* and a German version of *Carver and White's BIS/BAS* questionnaire, the latter being the most widely used questionnaire in RST research to date. The third section of the results reports a genetic validation of the new RST questionnaire, with a specific focus on the potential relation between BIS sensitivity and the AVPR1a gene.

### Section 1: psychometric analysis of *Reuter and Montag's rRST-Q*

In Table 3, means and standard deviations for the German version of *Reuter and Montag's rRST-Q* are provided, including descriptive statistics for the male and female participants separately.

**Table 3:** **Means and standard deviations for the full German sample and males and females separately for the different scales of *Reuter and Montag's rRST-Q* (data of the present study)**.

**Personality dimensions**	**Complete sample**	**Male sample**	**Female sample**	**Significant differences between males and females?**
rBAS	*M* = 2.89, *SD* = 0.45, *N* = 1814	*M* = 2.88, *SD* = 0.46, *N* = 704	*M* = 2.90, *SD* = 0.44, *N* = 1110	*F*_(1, 1800)_ = 0.74, *p* = 0.39
rBIS	*M* = 2.55, *SD* = 0.48, *N* = 1814	*M* = 2.41, *SD* = 0.46, *N* = 704	*M* = 2.65, *SD* = 0.47, *N* = 1110	*F*_(1, 1800)_ = 107.99, *p* < 0.001
FFFS	*M* = 2.32, *SD* = 0.39, *N* = 1814	*M* = 2.23, *SD* = 0.40, *N* = 704	*M* = 2.38, *SD* = 0.38, *N* = 1110	*F*_(1, 1800)_ = 64.58, *p* < 0.001
Fight	*M* = 2.68, *SD* = 0.50, *N* = 1814	*M* = 2.78, *SD* = 0.50, *N* = 704	*M* = 2.61, *SD* = 0.49, *N* = 1110	*F*_(1, 1800)_ = 51.50, *p* < 0.001
Flight	*M* = 2.40, *SD* = 0.55, *N* = 1803	*M* = 2.25, *SD* = 0.56, *N* = 703	*M* = 2.50, *SD* = 0.53, *N* = 1100	*F*_(1, 1800)_ = 93.70, *p* < 0.001
Freezing	*M* = 2.26, *SD* = 0.50, *N* = 1813	*M* = 2.23, *SD* = 0.53, *N* = 704	*M* = 2.28, *SD* = 0.48, *N* = 1109	*F*_(1, 1800)_ = 4.00, *p* < 0.01

The internal consistencies in terms of Cronbach's Alpha for BIS, BAS and FFFS were good in the German as well as in the English version. The rather low reliabilities for the FFFS subscales are due to the small number of items per scale (see Table 4). However, this is similar for the BAS subscales of the classic BIS/BAS questionnaire by Carver and White (see Table 5).

**Table 4:** **Internal consistencies (Cronbach's Alpha) for the scales of *Reuter and Montag's rRST-Q* in the German and English sample (data of the present study)**.

**Personality dimension**	**Number of items**	**German version**	**English version**
rBIS	11	0.78 (*n* = 1796)	0.76 (*n* = 297)
rBAS	8	0.77 (*n* = 1803)	0.74 (*n* = 295)
FFFS	12	0.75 (*n* = 1777)	0.75 (*n* = 291)
Fight	5	0.66 (*n* = 1797)	0.60 (*n* = 297)
Flight	3	0.53 (*n* = 1803)	0.55 (*n* = 296)
Freezing	4	0.55 (*n* = 1804)	0.52 (*n* = 296)

**Table 5:** **Internal consistencies (Cronbach's Alpha) for the *Carver and White BIS/BAS* scales in the German sample of the present study and the initial translation paper by Strobel et al. (2001)**.

**Personality dimension**	**Number of of items**	**German version (Reuter/Montag)**	**German version Strobel et al. (2001)**
BIS	7	0.71 (*n* = 1298)	0.78 (*n* = 295)
BAS	13	0.78 (*n* = 1306)	0.81 (*n* = 297)
BAS drive	4	0.69 (*n* = 1307)	0.69 (*n* = 290)
BAS fun seeking	4	0.58 (*n* = 1306)	0.67 (*n* = 296)
BAS reward responsiveness	5	0.55 (*n* = 1304)	0.69 (*n* = 296)

**Figure 1:**
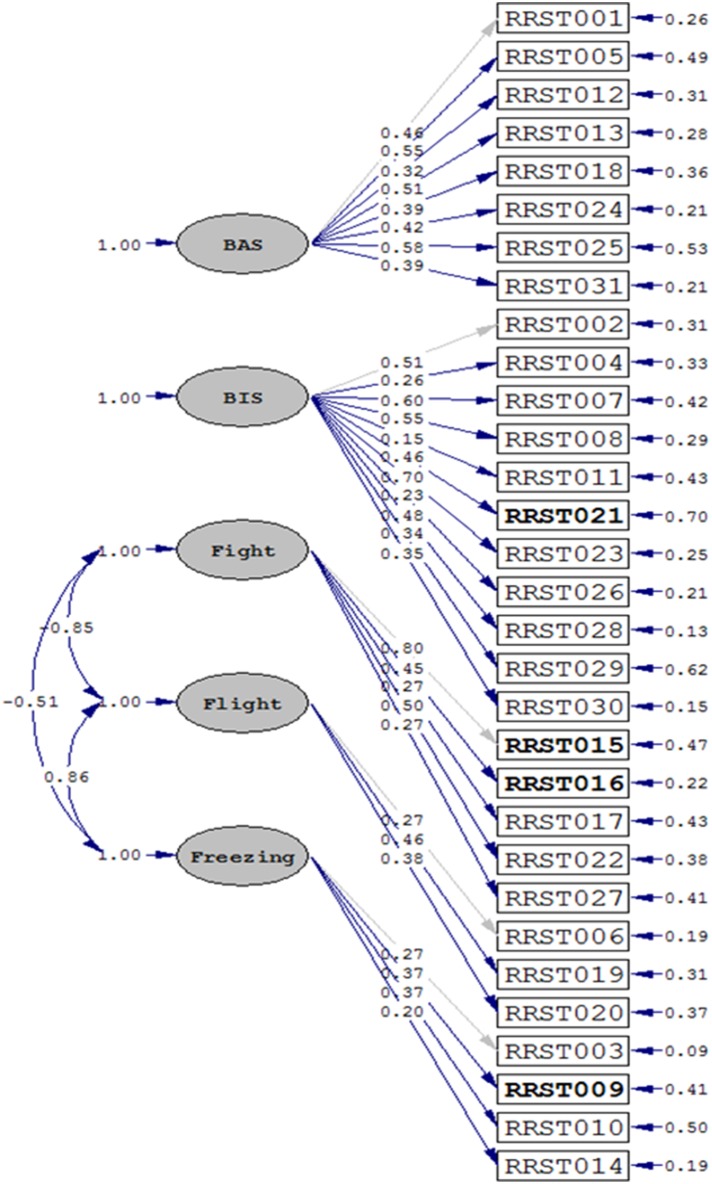
**Results for the CFA in the German sample (*N* = 1749)**. Fit indices were as follows: Chi^2^ = 4061.72, *df* = 431, *p* < 0.0001; RMSEA = 0.069; CFI = 0.92. A similar fit could be observed for the English sample (*N* = 286): Chi^2^ = 871.27, *df* = 431, *p* < 0.0001: RMSEA = 0.060; CFI = 0.93. Recoded items are marked in bold letters.

In order to test if the factor structure of the *Revised Reinforcement Sensitivity Theory Questionnaire (rRST-Q)* is in accordance with our theoretical assumptions, we ran CFAs using the LISREL software package (LISREL 8.80 by Jöreskog and Sorböm (1996); Science Software International, Inc). Given the ordinal nature of the questionnaire data (a 4-point Likert scale), the CFAs were based on polychoric covariance matrices and asymptotic covariance matrices. Parameter estimates were calculated using the Robust Diagonally Weighted Least Squares (DWLS) method. As indicated by the fit indices, the data showed good fit to our theoretical model in the German (Chi^2^ = 4061.72, *df* = 431, *p* < 0.0001; RMSEA = 0.069; CFI = 0.92; see Figure 1), and in the English sample (Chi^2^ = 871.27, *df* = 431, *p* < 0.0001: RMSEA = 0.060; CFI = 0.93).

### Section 2: associations between *Reuter and Montag's rRST-Q* and the *Carver and White BIS/BAS* scale

In Table 6, the inter-correlations between the *Reuter and Montag rRST-Q* dimensions and *Carver and White's BIS/BAS* scales are provided. Of note, the shared variance between the classic BAS from the Carver and White scale and the revised BAS from the new inventory is about 25%. Similarly, the shared variance between the classic BIS scale and its revised form was also around 25%. In Table 7 we include additional information on the correlations between the subscales of the FFFS, and the revised BIS and BAS scales; Table 8 provides correlations between the FFFS subscales and *Carver and White's BIS/BAS*.

**Table 6:** **Correlations between the *Carver and White BIS/BAS* scales and the *Reuter and Montag rRST-Q* (*N* = 1090)**.

	**BAS**	**BIS**	**rBAS**	**rBIS**	**FFFS**
BAS	1	*r* = 0.02,	*r* = 0.50,	*r* = −0.04,	*r* = −0.16,
		*p* = 0.61	*p* < 0.001	*p* = 0.16	*p* < 0.001
BIS		1	*r* = −0.28,	*r* = 0.45,	*r* = 0.45,
			*p* < 0.001	*p* < 0.001	*p* < 0.001
rBAS			1	*r* = −0.29,	*r* = −0.41,
				*p* < 0.001	*p* < 0.001
rBIS				1	*r* = 0.55,
					*p* < 0.001
FFFS					1

**Table 7:** **Correlations between the FFFS dimension and its subscales and the rBIS/rBAS (German sample; *N* = 1090)**.

	**FFFS**	**rBIS**	**rBAS**
Fight	*r* = −0.78, *p* < 0.001	*r* = −0.34, *p* < 0.001	*r* = 0.37, *p* < 0.001
Flight	*r* = 0.76, *p* < 0.001	*r* = 0.48, *p* < 0.001	*r* = −0.34, *p* < 0.001
Freezing	*r* = 0.69, *p* < 0.001	*r* = 0.47, *p* < 0.001	*r* = −0.23, *p* < 0.001

**Table 8:** **Correlations between Reuter and Montag's FFFS subscales and *Carver and White's BIS/BAS* scales (German sample; *N* = 1090)**.

	**Fight**	**Flight**	**Freezing**
BAS	*r* = 0.21, *p* < 0.001	*r* = −0.09, *p* = 0.003	*r* = −0.04, *p* = 0.20
BIS	*r* = −0.32, *p* < 0.001	*r* = 0.47, *p* < 0.001	*r* = 0.26, *p* < 0.001

### Section 3: analysis of the genetic variation of the AVPR1a gene in relation to the behavioral inhibition system

**Figure 2:**
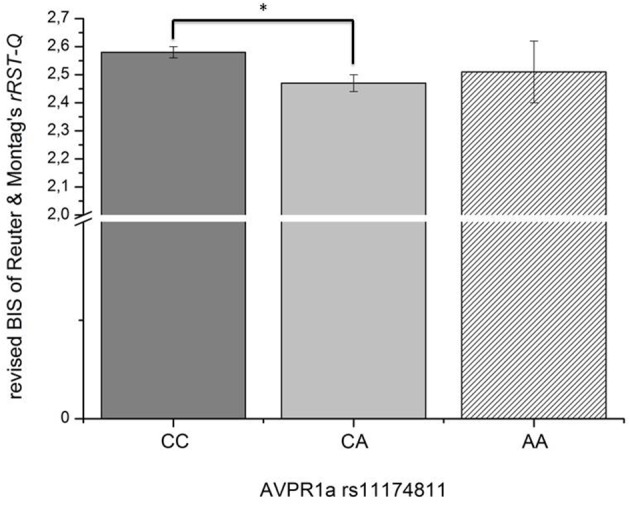
Association of genetic variation of the AVPR1a gene and anxiety measured with the revised BIS scale of Reuter and Montag rRST-Q (Means and SEMs are depicted; ^*^the contrast is significant at p = 0.002 level).

**Figure 3:**
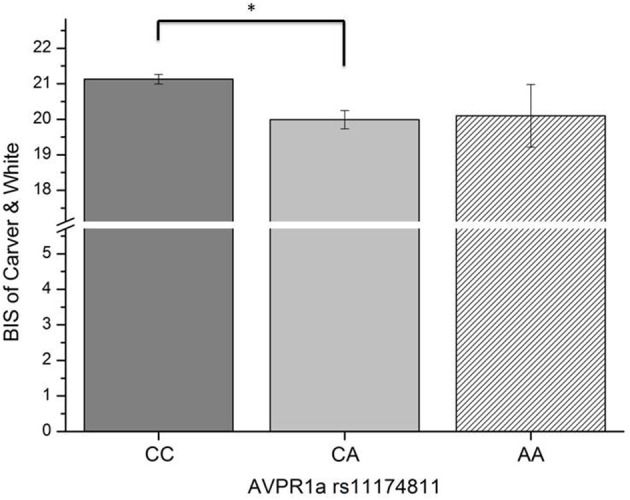
Association of genetic variation of the AVPR1a gene and anxiety measured with the BIS scale of Carver and White (Means and SEMs are depicted, ^*^contrast is significant at p < 0.001 level).

In this third section of the results, we explored the relation of the AVPR1a gene and its functional polymorphism rs11174811 with both the classic and revised BIS scales. From the total sample described above in Section 1 of the results, a subgroup of *n* = 1090 participants provided buccal swaps for genotyping rs11174811. The genotype distribution was as follows: CC = 840, CA = 230, AA = 20 (Hardy Weinberg Equilibrium: Chi^2^ = 0.84, df = 1, n.s.). A MANCOVA with the *Carver and White BIS/BAS* dimensions (and the BAS subscales) and with Reuter and Montag's scales revealed a significant effect of rs11174811 on both the classic and the revised BIS dimensions [*F*_(2, 1087)_ = 7.93, *p* < 0.001 vs. *F*_(2, 1087)_ = 5.03, *p* = 0.007, respectively]. As both of the BIS dimensions correlated with age (BIS: *r* = −0.14, *p* < 0.001 vs. rBIS: *r* = −0.19, *p* < 0.001), and with gender, with females having significantly higher scores [BIS: *F*_(1, 1088)_ = 143.30, *p* < 0.001 vs. rBIS: *F*_(1, 1088)_ = 69.15, *p* < 0.001], we undertook additional analyses, including gender as a second independent variable and age as a covariate. No gender by gene interaction effects could be observed on the BIS scales. The inclusion of age as a covariate did not change the significant influence of rs11174811 on the BIS. A *post-hoc* test revealed that the contrast for the genotypes CC vs. CA was significant. The group consisting of AA carriers was excluded from further interpretation at this point, because of the small group showing no clear trend in either the AA or AC direction (*n* = 20; see also Figures 2, 3). As shown in Tables 9, 10, no significant effect of rs11174811 could be detected on our measure of FFFS, nor on any of the other RST dimensions across both personality inventories.

**Table 9:** **Means and standard deviations of *Reuter and Montag's rRST-Q scales* depending on AVPR1a's rs11174811**.

**Personality dimensions**	**CC**	**CA**	**AA**	**Significant differences?**
rBAS	*M* = 2.87, *SD* = 0.45, *N* = 840	*M* = 2.92, *SD* = 0.51, *N* = 230	*M* = 2.91, *SD* = 0.37, *N* = 20	*F*_(2, 1087)_ = 1.28, *p* = 0.28
rBIS	*M* = 2.58, *SD* = 0.46, *N* = 840	*M* = 2.47, *SD* = 0.51, *N* = 230	*M* = 2.51, *SD* = 0.48, *N* = 20	*F*_(2, 1087)_ = 5.03, *p* = 0.007
FFFS	*M* = 2.35, *SD* = 0.39, *N* = 840	*M* = 2.30, *SD* = 0.40, *N* = 230	*M* = 2.37, *SD* = 0.32, *N* = 20	*F*_(2, 1087)_ = 1.57, *p* = 0.21
Fight	*M* = 2.66, *SD* = 0.50, *N* = 840	*M* = 2.69, *SD* = 0.50, *N* = 230	*M* = 2.68, *SD* = 0.31, *N* = 20	*F*_(2, 1087)_ = 0.47, *p* = 0.63
Flight	*M* = 2.44, *SD* = 0.55, *N* = 840	*M* = 2.40, *SD* = 0.56, *N* = 230	*M* = 2.53, *SD* = 0.56, *N* = 20	*F*_(2, 1087)_ = 0.88, *p* = 0.42
Freezing	*M* = 2.28, *SD* = 0.49, *N* = 840	*M* = 2.20, *SD* = 0.52, *N* = 230	*M* = 2.30, *SD* = 0.53, *N* = 20	*F*_(2,1087)_ = 2.10, *p* = 0.12

**Table 10:** **Mean and standard deviations of *Carver and White's BIS/BAS* scale depending on AVPR1a's rs11174811**.

**Personality dimensions**	**CC**	**CA**	**AA**	**Significant differences?**
BAS	*M* = 39.98, *SD* = 4.22, *N* = 840	*M* = 39.84, *SD* = 4.69, *N* = 230	*M* = 41.20, *SD* = 3.86, *N* = 20	*F*_(2, 1087)_ = 0.91, *p* = 0.40
BAS drive	*M* = 12.03, *SD* = 1.96, *N* = 840	*M* = 11.93, *SD* = 2.04, *N* = 230	*M* = 12.55, *SD* = 1.67, *N* = 20	*F*_(2, 1087)_ = 0.94, *p* = 0.39
BAS fun seeking	*M* = 11.59, *SD* = 1.96, *N* = 840	*M* = 11.51, *SD* = 2.08, *N* = 230	*M* = 12.10, *SD* = 1.89, *N* = 20	*F*_(2, 1087)_ = 0.85, *p* = 0.43
BAS reward responsiveness	*M* = 16.36, *SD* = 1.99, *N* = 840	*M* = 16.40, *SD* = 2.14, *N* = 230	*M* = 16.55, *SD* = 1.76, *N* = 20	*F*_(2, 1087)_ = 0.12, *p* = 0.89
BIS	*M* = 21.13, *SD* = 3.90, *N* = 840	*M* = 19.99, *SD* = 4.13, *N* = 230	*M* = 20.10, *SD* = 3.39, *N* = 20	*F*_(2, 1087)_ = 7.93, *p* < 0.001

## Discussion

This study had three key aims. First, we sought to develop a new self-report measure for the revised RST in order to better distinguish between aspects of personality concerned with fear and anxiety. The most widely used self-report measure in RST research, the *Carver and White BIS/BAS* scales, was developed under the classic model of RST, and the BIS scale in that measure arguably conflates processes related to the BIS and FFFS in the item content. Given the putative separation of the FFFS and the BIS in the revised RST in terms of behavioral functioning and their neuropsychopharmacological bases, self-report measures that seek to separate the FFFS and BIS are desirable. On that basis, and in line with revised RST, the new inventory attempts to disentangle the emotions of fear and anxiety by including separate scales for the revised BIS (reflecting anxiety) and for the FFFS (reflecting the emotion of fear). Of note, we designed the BIS scale to measure hesitation and cautious behavior in conflict situations, such as deciding between two (even potentially positive) options (e.g., “If I have the choice between two appealing options, I have difficulty deciding on one.”). As well as difficulties in behavioral choice, cognitions related to tolerance of uncertainty are also reflected in the revised BIS in our questionnaire (e.g., “I find it hard to bear uncertainty.”). The scale for the FFFS, measuring individual differences in fear tendencies, comprises the most important classes of behavioral fear responses, namely Fight, Flight, and Freezing. Finally, the BAS scale is designed to measure individual differences in reward-seeking, drive and energy (e.g., “I'm a spontaneous person.” or “Most of the time I have a thirst for action.”).

Despite some similarities in the conceptualization of the revised RST between the *RSQ* by Smederevac et al. (2014) and *Reuter and Montag's rRST-Q* there are also apparent differences. With respect to the BIS, the *rRST-Q* concentrates on conflicts without focusing on irrational interpretations of stimuli as the *RSQ* does. The conceptualization of the BAS is broader in the *rRST-Q* than in the *RSQ*: besides sensitivity to signals of reward, drive, energy and risk taking are also included.

The correlations between the dimensions within *Reuter and Montag's rRST-Q* show that the BAS is negatively associated with both the BIS and FFFS. As activation of the BAS is clearly associated with approach behavior or “wanting,” this is not surprising, as BIS activation reflects orienting and risk assessment behavior (e.g., careful approach behavior, which can switch to activation of the FFFS in the presence of more overt and physically closer threats—ergo, avoidance behavior). In line with this, both the BIS and the FFFS are positively correlated and can be positioned on the side of negative emotionality. Importantly, from a psychometric point of view, our new inventory shows good internal consistencies across the scales and good model fit when using CFA to model the latent variables of the questionnaire. It should be noted that the internal consistencies of the Flight and Freezing subscales are potentially a little lower than ideal, however they are each comprised of only several items, and so this may be expected.

The second aim of the study was to cross validate *Reuter and Montag's rRST-Q* with the *Carver and White BIS/BAS* scale. The results of this cross validation show that both the classic BAS and revised BAS, and also the classic BIS and revised BIS scale, correlate to about.50—hence 25% of the variance of these constructs overlap. This obviously also makes clear that a large portion of the variance does not overlap (75%), and so as a consequence the *Reuter and Montag's rRST-Q* are clearly measuring something related to yet distinct from the Carver and White dimensions. Future studies including both *Carver and White's BIS/BAS* scale and *Reuter and Montag's rRST-Q* are needed, particularly studies examining processes related to fear and anxiety in the context of revised RST, but also those studies examining BAS-related processes and functions. Establishing whether the *Reuter and Montag's rRST-Q* has incremental and/or divergent validity in relation to existing RST-related measures is clearly an important next step.

The final aim of this study was to examine individual differences of the BIS in relation to a genetic variation on the AVPR1a gene. In line with the previous literature, we showed that the gene coding for vasopressin 1a receptor is involved in human anxiety. Carriers of the CC variant of rs11174811 showed significantly elevated anxiety scores, measured in terms of Gray's Behavioral Inhibition System. As already described above, expression levels in homozygous C-allele carriers (genotype CC) have been reported to be significantly lower compared to carriers of at least one minor A-allele (genotypes AA or CA; Maher et al., 2011). As a consequence, a putatively lower number of vasopressin 1a receptors are associated with elevated anxiety levels, because the anxiety lowering effects of vasopressin (Appenrodt et al., 1998) cannot unfold completely due to lower binding possibilities. But: This interpretation would be against the findings from genetic animal research showing that knocking out the AVPR1a gene is associated with lower anxiety (Egashira et al., 2007).

Interestingly, rs11174811 showed a significant effect on both the BIS measured with the Carver and White scale, as well as on the revised BIS measured with *Reuter and Montag's rRST-Q*. Given the correlation of 0.45 between the classic BIS and the revised BIS shown above, the genetic variation of the AVPR1a gene clearly targets the shared variance of both constructs. How can this be explained? When comparing Carver and White's BIS and Reuter and Montag's BIS scale it is apparent that Carver and White's BIS is a little more multifaceted compared to our revised BIS scale. More specifically, Carver and White included a wide range of BIS items in their questionnaire, ranging from explicitly feeling anxious (e.g., item 2 of their scale), to restlessness when being confronted with an unpleasant event (item 16 of their scale). In contrast, the revised BIS scale of our newly designed questionnaire includes no item explicitly referring to feeling anxious. Instead, Reuter and Montag's revised BIS scale describes being unable to bear uncertainty or often being indecisive, which targets one major issue in the revised RST. From our point of view, the overlap between the scales (and the genetic effect targeting the shared variance) could possibly be explained by the aspect of restlessness when being confronted with an unpleasant event (in the Carver and White questionnaire), which is close to our concept of being indecisive or overly careful when confronted with uncertainty.

Importantly, the genetic effect of rs11174811 was only significant in the context of BIS sensitivity; no significant effect was observed on the FFFS scale, measuring individual differences in fear and avoidance tendencies, nor any other RST scales on either of the questionnaires administered. The genetic variant investigated in this study seems to target anxiety, but not fear, in terms of the conceptualization of *Reuter and Montag's rRST-Q*. This finding supports the divergent validity of the BIS and FFFS dimensions in the rRST-Q, a key aim in the development of this questionnaire, and is potentially of wider importance for the revised model of RST, in terms of identifying neurobiological markers that reliably distinguish between trait measures of these constructs. Clearly, the genetic finding in this study represents a beginning point in this process, but an important beginning point nonetheless.

It should be noted that there are existing attempts to develop self-report measures in the context of revised RST (e.g., the “Jackson-5”; Jackson, 2009), and also attempts to modify the psychometric structure of measures designed under the classic RST in line with revised RST. For example, Heym et al. (2008) suggested the original Carver and White BIS scale could be decomposed into separate BIS and FFFS dimensions, based on an evaluation of the item wording and results of confirmatory factor analysis. Despite this, the research so far using these new or modified measures has tended to focus on validation using other psychometric self-report measures, laboratory-based behavioral tasks or “real-world” behaviors, and there has been limited research on the neurobiological markers associated with these new scales, particularly in terms of separating the BIS and FFFS. Thus, the genetic data reported in this study represents a relatively novel and important step in this endeavor.

## Conclusion

*Reuter and Montag's rRST-Q* is a new self-report measure, developed in line with theoretical assumptions derived from Gray and McNaughton's RST (2000). The psychometric properties of the scale, including its factorial structure and internal consistencies were supported in both a German and English language version of the measure. Correlations between *Reuter and Montag's rRST-Q* and an existing RST measure, the *Carver and White BIS/BAS* scales, showed that the new scale dimensions correlated in the expected direction with the Carver and White dimensions, but the correlations were not large enough to suggest high redundancy in the new dimensions. A first validation study using a molecular genetic approach found a significant association between a functional polymorphism on the AVPR1a gene (rs11174811) and the BIS. The genetic association was shown with respect to the BIS dimension in both the *Carver and White BIS/BAS* questionnaire and in the *rRST-Q*. Further, the genetic association was not shown for the FFFS dimension in the rRST-Q, supporting the divergent validity of the BIS and FFFS dimensions in this scale, and highlighting a potentially useful genetic marker that could be used to evaluate existing or new measures developed under the revised RST. This study should clearly be seen as a first step in the validation of this new revised RST measure. In particular, no validation of the revised BAS scale was attempted in this study, and this may be a focus for future work on this scale. More broadly, future studies will be needed to search for further genetic, endocrinological and brain imaging validation of this new inventory. In addition, this new tool will also need to be further evaluated in relation to other self-report measures and using theoretically relevant behavioral and experimental laboratory tasks.

### Conflict of interest statement

The authors declare that the research was conducted in the absence of any commercial or financial relationships that could be construed as a potential conflict of interest.

## Footnotes

^1^The important distinction between the emotions of fear and anxiety came with revised RST and will be discussed in the light of withdrawal/avoidance behavior further on.

^2^The FFFS was already included in the original version of the RST as the so called FFS and was activated by unconditioned unpleasant stimuli.

^3^BIS correlates with Harm Avoidance and Neuroticism at about 0.55–0.59 (Montag et al., 2013).

^4^These two genes code for the vasopressin 1a or 1b receptors. Of note, the gene coding for the 1b receptor has not been the major focus of research until now.
